# Complete nucleotide sequence of pRS218, a large virulence plasmid, that augments pathogenic potential of meningitis-associated *Escherichia coli* strain RS218

**DOI:** 10.1186/s12866-014-0203-9

**Published:** 2014-08-28

**Authors:** Dona Saumya S Wijetunge, Kurundu Hewage Eranda M Karunathilake, Atul Chaudhari, Robab Katani, Edward G Dudley, Vivek Kapur, Chitrita DebRoy, Subhashinie Kariyawasam

**Affiliations:** Department of Veterinary and Biomedical Sciences, Pennsylvania State University, 16802 University Park, PA USA; Department of Food Science, Pennsylvania State University, 16802 University Park, PA USA; Center for Molecular Immunology and Infectious Disease, 16802 University Park, PA USA

**Keywords:** DNA sequence, *Escherichia coli*, Neonates, Meningitis, Plasmid, Virulence

## Abstract

**Background:**

*Escherichia coli* is the most predominant Gram-negative bacterial pathogen associated with neonatal meningitis. Previous studies indicated that the prototypic neonatal meningitis *E. coli* (NMEC) strain RS218 (O18:K1:H7) harbors one large plasmid. Objectives of the present study were to analyze the complete nucleotide sequence of this large plasmid (pRS218) and its contribution to NMEC pathogenesis using *in vitro* and *in vivo* models of neonatal meningitis.

**Results:**

The plasmid is 114,231 bp in size, belongs to the incompatibility group FIB/IIA (IncFIB/IIA), and contains a genetic load region that encodes several virulence and fitness traits such as enterotoxicity, iron acquisition and copper tolerance. The nucleotide sequence of pRS218 showed a 41- 46% similarity to other neonatal meningitis-causing *E. coli* (NMEC) plasmids and remarkable nucleotide sequence similarity (up to 100%) to large virulence plasmids of *E. coli* associated with acute cystitis. Some genes located on pRS218 were overly represented by NMEC strains compared to fecal *E. coli* isolated from healthy individuals. The plasmid-cured strain was significantly attenuated relative to the RS218 wild-type strain as determined *in vitro* by invasion potential to human cerebral microvascular endothelial cells and *in vivo* by mortalities, histopathological lesions in the brain tissue, and bacterial recovery from the cerebrospinal fluid of infected rat pups.

**Conclusions:**

The pRS218 is an IncFIB/IIA plasmid which shares a remarkable nucleotide sequence similarity to large plasmids of *E. coli* associated with cystitis. Both *in vitro* and *in vivo* experiments indicated that pRS218 plays an important role in NMEC pathogenesis.

## Background

Neonatal meningitis (NM) and sepsis is the third most common disease in neonates that accounts for approximately 393,000 deaths per year worldwide [1]. *Escherichia coli* has been identified as the most predominant Gram-negative pathogen associated with NM [2–5]. Despite advanced antimicrobial therapy and supportive care, mortality and morbidity rates of NM due to neonatal meningitis-associated *E. coli* (NMEC) continue to be as high as 30-50% [6]. Other than high mortality, adverse consequences such as mental retardation, vision loss or impairment, hearing impairment and speech impediment of NM in surviving neonates are also a major medical concern [7,8].

Plasticity of *E. coli* genomes has led to the development of different pathovars of *E. coli* each of which is associated with a particular form of animal and/or human disease [9,10]. Genomic plasticity of *E. coli* is mainly due to the acquisition of ‘genomic islands’ through horizontal gene transfer by means of plasmids, phages and insertion sequences (IS) [9]. Of these elements, bacterial plasmids are self-replicating extra-chromosomal genetic materials which have the potential to transmit a variety of phenotypic characteristics among the same or different species of bacteria [9–11]. These phenotypic characteristics include novel metabolic capabilities, antibiotic resistance, heavy metal tolerance, virulence traits that are important for bacterial adherence, invasion and survival in host tissues [10,11]. Plasmid that encodes such phenotypic characteristics may provide competitive advantages to the bacterium for survival and adaptation to novel niches.

Many virulence associated plasmids have been identified in pathogenic *E. coli* [10,12–14]. A vast majority of these plasmids belong to IncF compatibility group. Structurally, IncF plasmids consist of a conserved region common to all IncF plasmids which encodes conjugal transfer proteins, replication proteins and plasmid stability proteins and a ‘genetic load region’ or a variable region that encodes various virulence and fitness traits. A recent study that analyzed over 40 completed genomic sequences of IncF plasmids of *E. coli* revealed that these plasmids have evolved as virulence plasmids by acquiring novel virulence traits to their ‘genetic load regions’ through IS-mediated site specific recombination [10]. Also, comparative genomic analysis of virulence plasmids in each pathovar of *E. coli* has shown that these genetic load regions encode virulence traits that are essential for and specific to their respective pathotype [10]. These data suggest that acquisition of plasmid-encoded genes may play a significant role in the emergence of pathogens and different pathotypes of *E. coli*.

Although many virulence-associated plasmids in various intestinal pathogenic *E. coli* have been sequenced and studied, only a few virulence plasmids associated with each pathotype of extra-intestinal pathogenic *E. coli* (ExPEC) causing human infection have been sequenced [10]. For example, at the time of preparing this manuscript, only two plasmid sequences from NMEC strains were available in the public domain [14,15]. These two strains represent two of three major serogroups of *E. coli* (O18, O45 and O7) that have been implicated in NM; pECOS88 from *E. coli* S88 (O45:K1) and pEC10A-D from *E. coli* CE10 (O7:K1). Despite the fact that the NMEC prototypic strain RS218 belonging to O18 serogroup is the most commonly used *E. coli* strain to study NMEC pathogenesis since 1980’s, its genomic sequence including the plasmid, has not been reported [16]. It has been documented that the NMEC RS218 strain harbors a large plasmid and similar sized plasmids have been observed in other NMEC and avian pathogenic *E. coli* (APEC) belonging to the O18 serogroup [17,18].

Therefore, the objectives of the present study were to: (i) analyze the nucleotide sequence of pRS218 and its genetic and evolutionary relationship with virulence-associated plasmids in other pathogenic *E. coli*, (ii) analyze the distribution of pRS218 genes among NMEC, and (iii) evaluate the contribution of pRS218 to NMEC pathogenesis by comparing the virulence of plasmid-cured and wild-type strains *in vitro* and *in vivo*.

## Results

### General properties of pRS218

**Figure 1:**
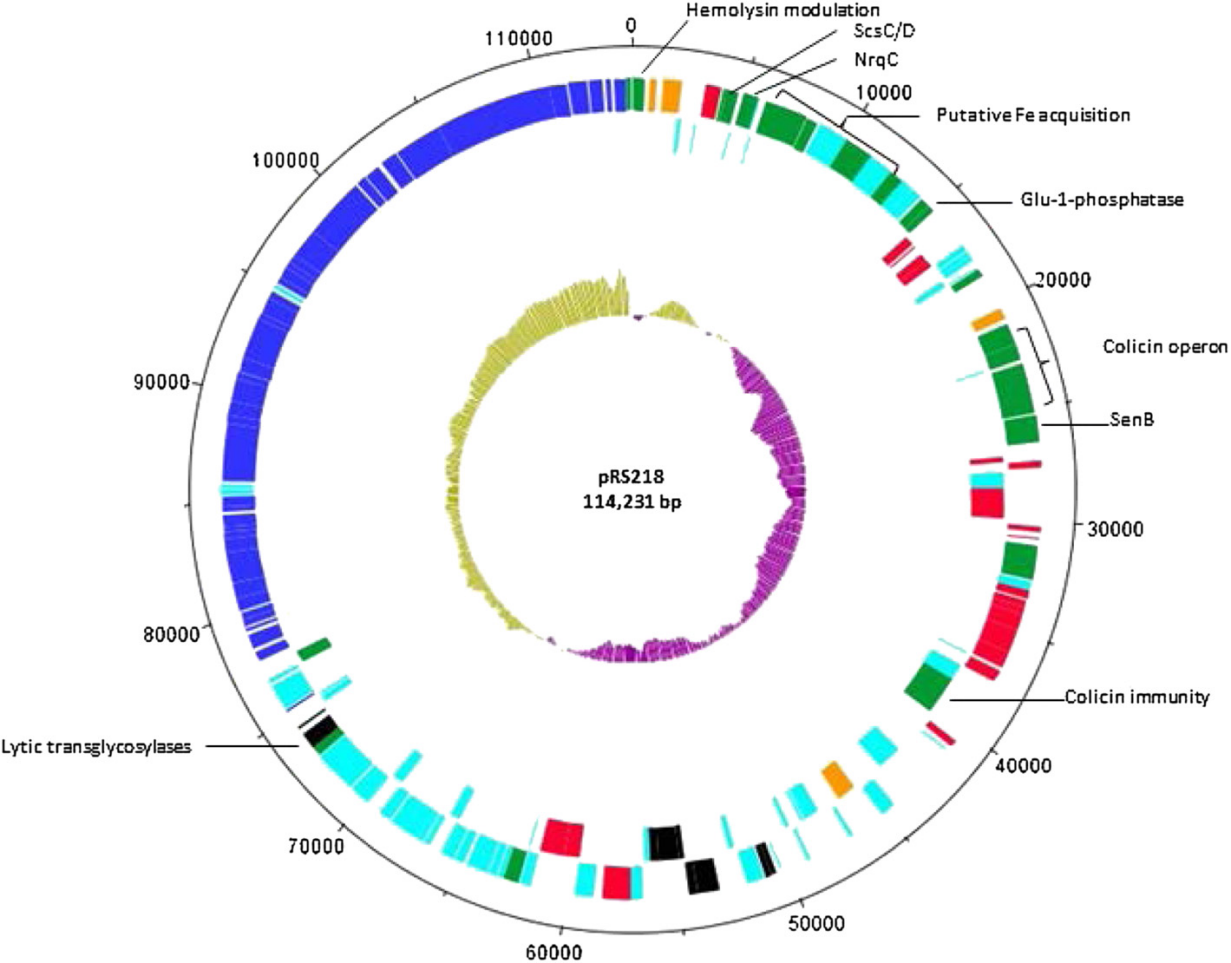
**Graphical circular map of pRS218.** From outside to the center: ORFs in forward strand, ORFs in reverse strand, and GC skew. Plasmid genes are color coded as follows: Blue, conjugal transfer genes; Green, virulence or fitness-associated genes; Orange, plasmid replication genes; Red, IS elements; Black, plasmid stability genes; Light blue, hypothetical and putative genes. In the GC skew lime indicates the areas where the GC skew above average (51%) and purple indicates the areas below average.

Initial *de novo* assembly of short reads generated with Ion Torrent PGM technology identified 26 plasmid contigs ranging from 253 to 7,521 bp in length. These contigs were aligned to the reference plasmid sequence pUTI89 of uropathogenic *E. coli* strain UTI89 which was selected as the reference according to the sequence similarity of contigs (>90%). Complete sequence of pRS218 revealed that it is a circular plasmid of 114,231 bp in size with a G + C content of 51.02% (Figure 1). A total of one hundred and sixty open reading frames (ORFs) were annotated including IncFIB and FIIA replicons. Based on the blast analysis, nearly one third of the ORFs (n = 51) represents the genes involved in plasmid replication and conjugal transfer, along with 20 and 7 genes encoding mobile genetic elements (MGEs) and products involved in DNA repair, respectively. Of the remaining ORFs, 59 encode unknown or hypothetical proteins, and 23 represent genes previously characterized in other bacteria. The plasmid does not harbor any antibiotic resistance genes that may provide a selective advantage in the face of antibiotic therapy. Genetic load region of the pRS218 encodes several virulence- and fitness-associated genes which have been reported in other bacteria (Table 1). The annotated sequence of pR218 was deposited in GenBank at the NCBI [GenBank: CP007150].

**Table 1:** **Virulence and fitness-associated genes located on the genetic load region of pRS218**

**Gene name**	**Virulence/fitness-associated traits**	**Function**	**Reference**
pRS218_010	Putative Na-translocating NADH dehydrogenase	Na^+^efflux (NrqC subunit)	[19]
pRS218_013	High affinity Fe^2+^permease	Iron acquisition	[13]
pRS218_014	High affinity Fe^2+^periplasmic component	Iron acquisition	[13]
pRS218_015	High affinity Fe^2+^ protein, membrane component	Iron acquisition	[13]
pRS218_016	High affinity Fe^2+^ binding protein, permease	Iron acquisition	[13]
pRS218_017	Putative ABC transport sys, permease	Type I secretion	[13]
pRS218_018	Putative ABC transport system, ATP-binding	Type I secretion	[13]
pRS218_019	TonB-dependent heme/hemoglobin receptor	Iron acquisition	[13]
pRS218_039	SenB	Enterotoxin in EIEC/*Shigella*	[26]
pRS218_001	Putative GTP binding protein, YihA	Cell signaling and membrane ruffling	[20]
pRS218_190	Hemolysin expression modulating protein	Thermo-osmotic regulation of hly	[13]
pRS218_007	Suppressor for copper sensitivity ScsC/ScsD	Copper tolerance	[25]
pRS218_022	Glucose-1-phosphatase	Virulence regulator	[21]
pRS218_116	Lytic transglycosylases	Cell division	[22]

### pRS218 is remarkably similar to plasmids of *E. coli* strains that cause cystitis

**Figure 2:**
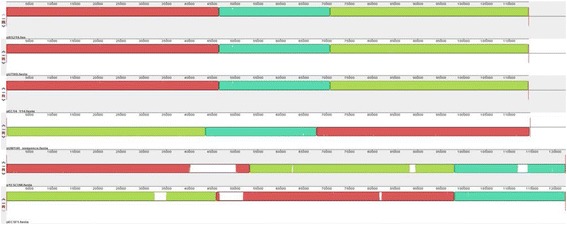
**Comparison of pRS218 sequence to some virulence plasmids of other** ***E. coli*** **.** Each code indicates a plasmid sequence. From top to bottom; pRS218, pUTI89 (a plasmid of the acute cystitis causing *E. coli* strain UTI89), pEC14_114 (a plasmid of the uropathogenic *E. coli* strain EC14), pUM146 (a plasmid of the adherent invasive *E. coli* strain UM146), p1ESCUM (a plasmid of the acute cystitis causing *E. coli* strain UMN026) and pECSF1 (a plasmid of the commensal *E. coli* strain SE15). Each color box indicates clusters of ortholog genes present in plasmid sequences. White spaces in the blocks indicate the sequences that are not present in other plasmid sequences.

**Figure 3:**
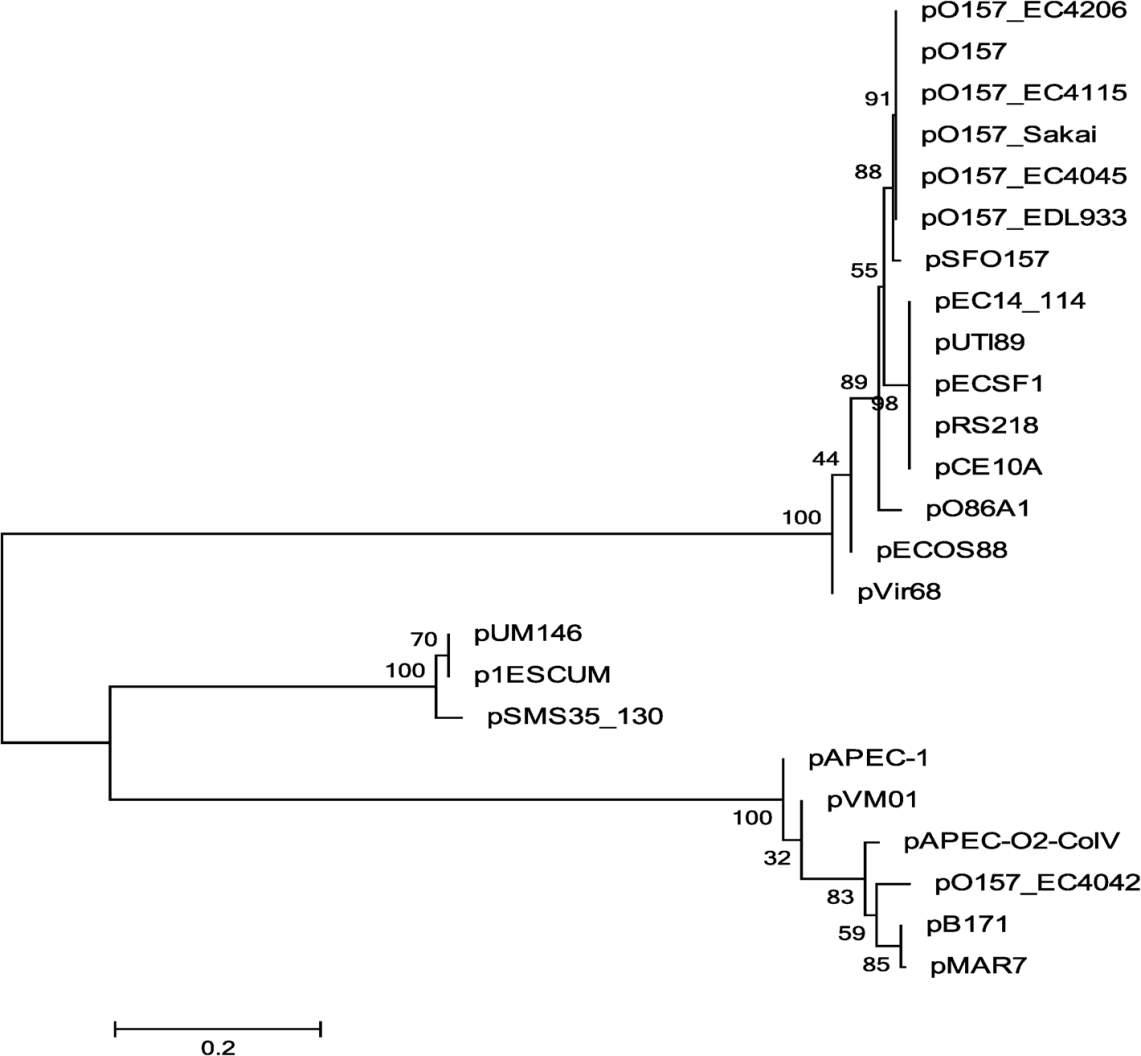
**Evolutionary relationship of IncFIB/IIA plasmids in pathogenic** ***E. coli*** **based on the repA1 sequence.** The percentage of replicate trees in which the associated taxa clustered together in the bootstrap test (500 replicates) is shown next to the branches.

The BLAST nucleotide algorithm (blastn) showed that pRS218 is 99% identical to plasmids pUTI89 [GenBank:CP000244], p1ESCUM [GenBank:CU928148] and pEC14_114 [GenBank:GQ398086] of *E. coli* causing acute cystitis, pUM146 [GenBank:CP002168] of a strain of *E. coli* associated with Crohn’s disease, and pECSF1[GenBank:AP009379] of an *E. coli* strain belonging to the phylogenetic group B2 which was isolated from feces of a healthy adult (Figure 2) [23]. Analysis of the *repA1* sequence of FIIA replicon of 24 IncFIB/IIA plasmids in pathogenic *E. coli* revealed three main lineages of virulence plasmids (Figure 3). All NMEC virulence plasmids were clustered into one lineage based on the *repA1* sequence suggesting a common origin. Interestingly, pRS218 showed an identical origin with several virulence plasmids of *E. coli* causing cystitis (pUTI89 and pEC14_114), pECSF1 of the commensal phylogenetic group B2 *E. coli* strain SE15 and pCE10A of NMEC strain CE10.

### Genes of pRS218 are overly represented in NMEC strains compared to fecal *E. coli*

Plasmid profiling revealed 27 of 53 (51%) of NMEC strains examined in the study harbored a plasmid similar in size to pRS218 (130-100 kb) (Table 2). Furthermore, PCR analysis revealed that a vast majority of pRS218-associated genes tested (n = 59) were overly represented (n = 52) among NMEC strains as compared to commensal *E. coli* (Table 3).

**Table 2:** **O serogroups of neonatal meningitis causing** ***E. coli*** **strains carrying pRS218-like plasmids**

**Isoate #**	**O serogroup**	**Plasmids similar in size to pRS218 +, present; -, absent**
NMEC1	18	-
NMEC2	75	-
NMEC3	2	-
NMEC4	Multiple	+
NMEC5	25	-
NMEC6	15	-
NMEC7	8	-
NMEC8	11	+
NMEC9	Negative	+
NMEC10	92	+
NMEC11	Negative	+
NMEC12	18	+
NMEC13	6	-
NMEC14	8	-
NMEC15	12,16	+
NMEC16	1	+
NMEC17	12,16	+
NMEC18	6	+
NMEC19	Multiple	+
NMEC20	8	-
NMEC21	19	-
NMEC22	18	+
NMEC23	75	-
NMEC24	107	-
NMEC25	1	+
NMEC26	8	+
NMEC27	8	+
NMEC28	Multiple	+
NMEC29	16	+
NMEC30	5	-
NMEC31	8	-
NMEC32	75	-
NMEC33	2	+
NMEC34	16	-
NMEC35	2	+
NMEC36	Multiple	-
NMEC37	21	-
NMEC38	1	+
NMEC39	1	+
NMEC40	1	+
NMEC41	18	-
NMEC42	18	-
NMEC43	7	+
NMEC44	78	+
NMEC45	25	-
NMEC46	1	+
NMEC47	1	-
NMEC48	1	-
NMEC49	Negative	-
NMEC50	1	-
NMEC51	18	+

**Table 3:** **Prevalence of pRS218 genes among neonatal meningitis causing** ***E. coli*** **and fecal commensal** ***E. coli*** **strains**

**Gene name**	**Predicted function**	**NMEC %**	**FEC %**	**Chi squire value**	**P value**	**Related pUTI89 locus**
pRS218_007	Copper sensitivity	98.11	46.94	65.229	<0.0001	P007
pRS218_008	Copper sensitivity	96.23	22.45	113.187	<0.0001	P008
pRS218_010	Na + traslocation	100.00	18.37	133.182	<0.0001	P009
pRS218_013	Iron permease	98.11	28.57	105.105	<0.0001	P010
pRS218_014	Iron transport	100.00	57.14	51.864	<0.0001	P011
pRS218_015	Membrane protein	96.23	18.37	124.113	<0.0001	P012
pRS218_016	ABC transporter	100.00	24.49	117.051	<0.0001	P013
pRS218_017	Membrane protein	94.34	77.55	12.706	0.0004	P014
pRS218_018	ABC transporter	98.11	55.10	51.425	<0.0001	P015
pRS218_019	Putative thioredoxin precursor	83.02	18.37	20.529	<0.0001	P016
pRS218_020	Hypothetical protein	100.00	18.37	133.182	<0.0001	P017
pRS218_022	Glucose-1-phosphatase	100.00	75.51	24.428	<0.0001	P018
pRS218_023	Glucose-1-phosphatase	98.11	16.33	137.169	<0.0001	P018
pRS218_031	Hypothetical protein	98.11	26.53	107.541	<0.0001	P024
pRS218_034	Colicin immunity	84.91	91.84	2.407	0.1208	P023
pRS218_035	ColicinJ production	66.04	100.00	49.668	<0.0001	P027
pRS218_036	ColicinJ production	77.36	97.96	20.16	<0.0001	P028
pRS218_038	ColicinJ production	100.00	26.53	112.012	<0.0001	P029
pRS218_039	Enterotoxin	100.00	71.43	33.918	<0.0001	P030
pRS218_042	Hypothetical protein	98.11	44.90	68.924	<0.0001	P034
pRS218_056	Hypothetical protein	100.00	6.12	177.358	<0.0001	P042
pRS218_057	ColicinJ production	100.00	100.00	0	1	P043
pRS218_060	Hypothetical protein	96.23	10.20	148.454	<0.0001	P045
pRS218_063	Hypothetical protein	100.00	24.49	120	<0.0001	P051
pRS218_064	Hypothetical protein	100.00	0.00	197.04	<0.0001	P052
pRS218_073	Hypothetical protein	94.34	53.06	43.152	<0.0001	P060
pRS218_074	Stability protein StbA	90.57	20.41	102.055	<0.0001	P062
pRS218_079	Hypothetical protein	98.11	22.45	120.333	<0.0001	P042
pRS218_080	Unknown	100.00	100.00	0	1	P065
pRS218_082	Hypothetical protein	100.00	34.69	96.296	<0.0001	P068
pRS218_083	Transposase	98.11	22.45	120.333	<0.0001	P071
pRS218_086	Hypothetical protein	98.11	22.45	120.333	<0.0001	P072
pRS218_088	Adenine-specific methyltransferase	100.00	13.33	151.027	<0.0001	P074
pRS218_089	Cytoplasmic protein	83.02	73.47	2.914	0.0878	P075
pRS218_090	Hypothetical protein	30.19	48.98	7.553	0.006	P076
pRS218_091	Hypothetical protein	98.11	55.10	51.425	<0.0001	P078
pRS218_091	Hypothetical protein	100.00	36.73	91.971	<0.0001	P078
pRS218_092	Putative antirestriction protein	73.58	83.67	3.014	0.0826	P079
pRS218_093	Phage protein MubC	100.00	81.63	16.986	<0.0001	P080
pRS218_094	Hypothetical protein	98.11	57.14	48.201	<0.0001	P081
pRS218_095	Hypothetical protein	75.47	6.12	98.786	<0.0001	P083
pRS218_099	Hypothetical protein	90.57	34.69	67.267	<0.0001	P088
pRS218_100	Hypothetical protein	100.00	34.69	96.296	<0.0001	P089
pRS218_105	Cytoplasmic protein	75.47	93.88	13.781	0.0002	P093
pRS218_106	Hypothetical protein	96.23	32.65	86.669	<0.0001	P094
pRS218_107	Adenine-specific methyltransferase	100.00	32.65	100.086	<0.0001	P095
pRS218_109	Hok/Gef cell toxic protein	100.00	93.88	0	0.9944	P097
pRS218_110	Hypothetical protein	98.11	26.53	107.541	<0.0001	P099
pRS218_113	Hypothetical protein	100.00	83.67	17.391	<0.0001	P100
pRS218_113	Hypothetical protein	100.00	73.47	31.214	<0.0001	P100
pRS218_114	Unknown	100.00	44.90	72.93	<0.0001	P101
pRS218_116	X polypeptide	97.96	46.94	65.229	<0.0001	P102
pRS218_118	TraJ/conjugal transfer	43.40	10.20	27.955	<0.0001	P104
pRS218_131	Hypothetical protein	100.00	93.88	6.186	0.0129	P116
pRS218_136	TraU/conjugal transfer	100.00	42.86	79.72	<0.0001	P120
pRS218_154	TraI/conjugal transfer	81.13	53.06	17.73	<0.0001	P138
pRS218_156	Dienelactone hydrolase	90.57	73.47	20.195	<0.0001	P141
pRS218_159	Hypothetical protein	90.57	93.88	1.087	0.2971	P144
pRS218_190	Hemolysin expression modulating protein	90.57	12.24	124.932	<0.0001	P145

*P* < 0.05 indicates a statistical significance.

### Plasmid-cured strain demonstrated a marked attenuation *in vitro* and *in vivo*

**Figure 4:**
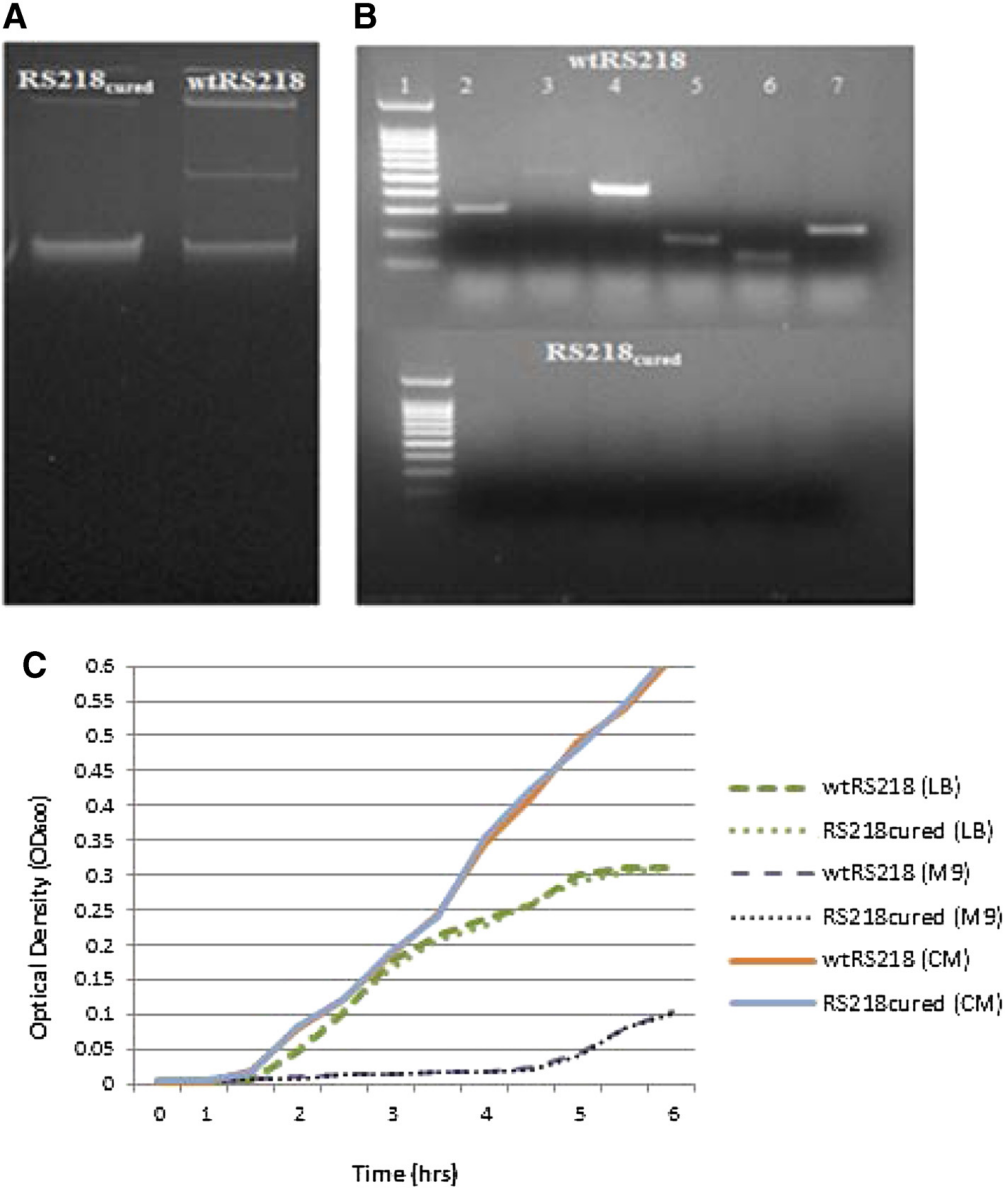
**Confirmation of pRS218 curing. A**, Plasmid profiles of wtRS218 and RS218_cured._ **B**, PCR amplification of selected pRS218 genes in wtRS218 and RS218_cured_. Lane 1,100 bp ladder; Lane 2, *senB*; Lane 3, *scsD*; Lane 4, *transposase*; Lane 5, *traU*; Lane 6, *pRS218_113*; Lane 7, *ycfA;* Lane 8, *ompA*. **C**, Growth of wtRS218 and RS218_cured_ *E. coli* in LB broth, M9 medium containing 10 μg/ml niacin broth (M9), and complete cell culture medium (CM).

**Figure 5:**
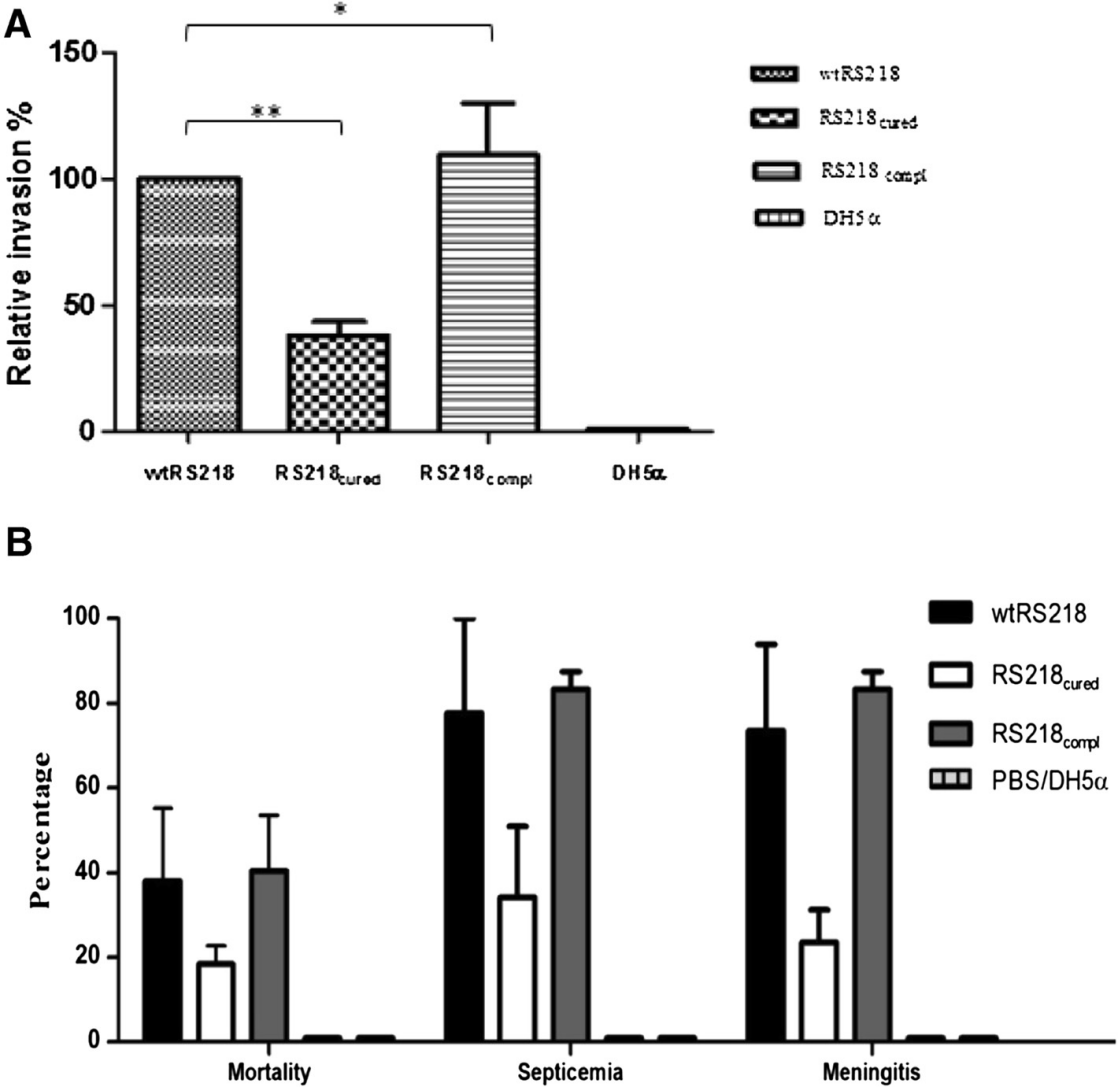
**Evaluation of virulence potential of pRS218** ***in vitro*** **and** ***in vivo.*** **A**, Involvement of pRS218 in invasion of hCMEC cells. **B**, Comparison of mortality, septicemia and meningitis among the groups of rat pups infected with wtRS218, RS218_cured_, RS218_compl_. ** denotes statistical significance and * denotes no statistical significance.

**Figure 6:**
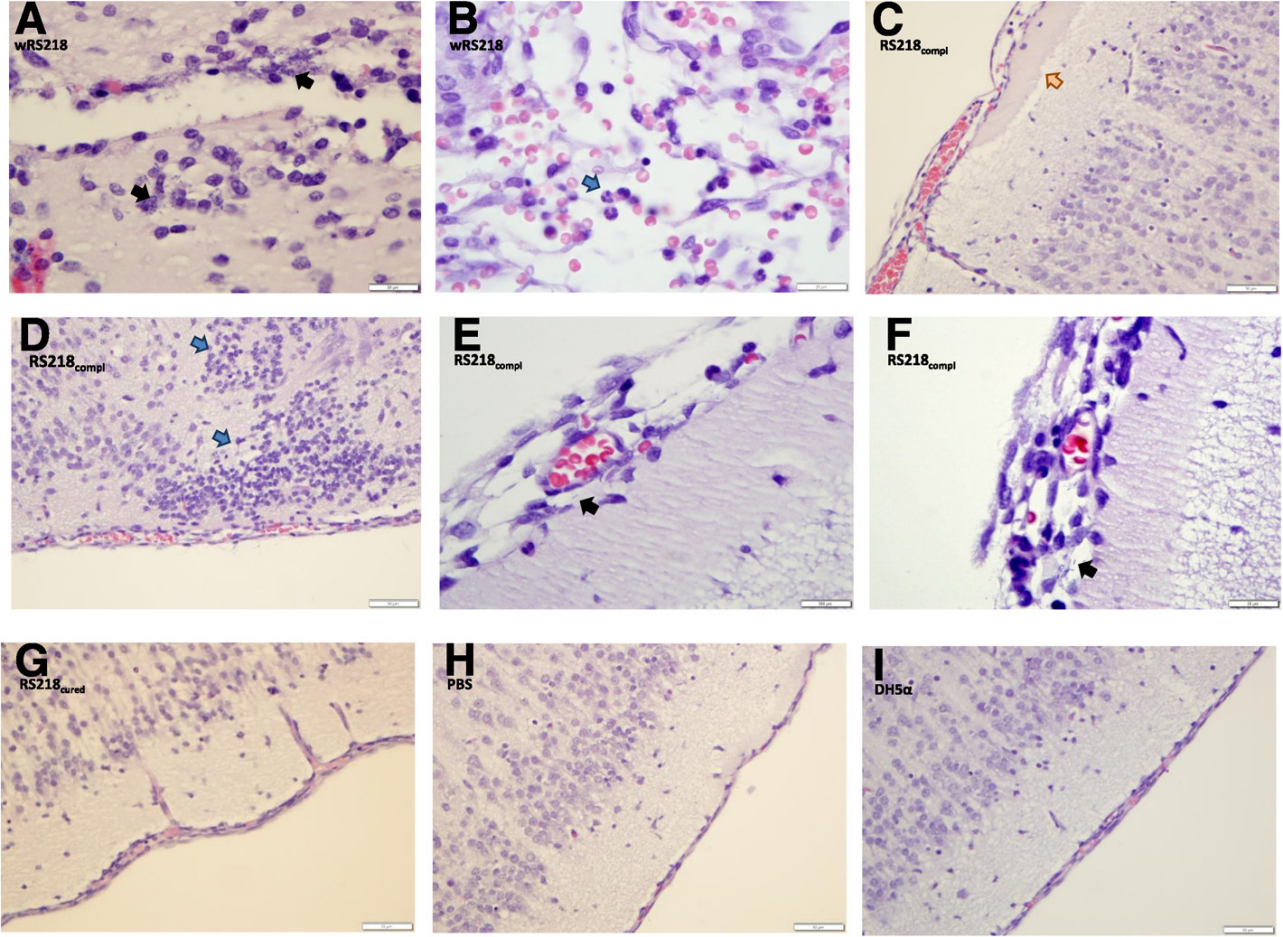
**Histopathological evaluation of brain tissue from rat pups.** Five-day-old rat pups were infected by the IP route with wtRS218, RS218_compl_, RS218_cured_, *E. coli* DH5α or PBS. Pups that survived were euthanized 24 hrs post-infection, and the brains were excised, embedded in formalin, sectioned in paraffin, and stained with haematoxylin and eosin. **A-F**: meningitic lesions observed in pups infected with wtRS218 **(A and B)** or RS218_compl_ **(C, D, E, and F)**. Arrows indicate rod-shaped bacteria in meninges and brain tissue (black), neutrophilic infiltration/neutrophilia (blue), and cerebral edema (orange). **G to I**: normal histology of brain tissue from pups inoculated with RS218_cured_ **(G)**, PBS **(H)** or DH5α **(I)**.

To analyze the virulence potential of pRS218, the plasmid was cured from the wild type strain by mutating *stbA* followed by 10% SDS treatment. Curing of plasmid was confirmed by the absence of the plasmid in the purified plasmid preparation and the absence of 5 selected genes of pRS218 by PCR in a crude DNA extract made from the plasmid-cured strain (RS218_cured)_. Figures 4A and B show the plasmid profiles and PCR amplification results of wild-type RS218 (wtRS218) and plasmid-cured RS218 (RS218_cured_). No difference was observed in the growth rates between wtRS218 and RS218_cured_ (Figure 4C). Virulence potential of pRS218 was determined by comparing RS218_cured_ with wtRS218 based on their ability to invade human cerebral microvascular endothelial (hCMEC/D3) cells *in vitro* and to cause septicemia, meningitis and mortality *in vivo* in a rat pup model of neonatal meningitis. *In vitro* invasion assays using hCMEC/D3 cells revealed a significant attenuation (p < 0.05) of RS218_cured_ (relative invasion 38 ± 9.6%) as compared to the wild type strain (100%) (Figure 5A). Furthermore, invasiveness was restored after complementation of RS218_cured_ strain with the pRS218 indicating its contribution to NMEC pathogenesis. Similar results were observed with the *in vivo* experiments as well. Although fewer pups died within 24 hrs post-infection in the groups infected with RS218_cured_ as compared to the groups infected with wtRS218 and RS218_compl_, there was no statistically significant difference in mortality rates between the three groups (Figure 5B). No mortalities were detected in the negative control group treated with PBS or *E. coli* DH5α. In groups infected with wtRS218 or RS218_compl_, 84-87% of rat pups that survived 24 hrs post-infection showed septicemia, whereas in groups treated with RS218_cured_ strain, only 33% had septicemia. In all three groups the number of bacteria in the blood was too numerous to count (>1.5-2.8 *10^4^ CFU/ml). Also, *E. coli* were re-isolated from CSF collected from 84-87% of rat pups infected with wtRS218 or RS218_compl_ whereas only 29% CSF samples collected from rat pups infected with RS218_cured_ strain contained *E. coli* suggesting a role of pRS218 in translocation of bacteria through the blood brain barrier (BBB) to cause meningitis. Similarly, histopathological evaluation of brain tissue from the rat pups inoculated with wRS218 or RS218_compl_ strains demonstrated lesions consistent with meningitis (Figure 6). The bacterial loads in CSF were 4.57 + 3.02*10^3^ in rat pups infected with wtRS218 strain and 3.77 + 2.24*10^3^ in rat pups infected with RS218_cured_ strain.

## Discussion

Virulence plasmids in bacterial pathogens have been shown to play a major role in pathogenesis of many bacterial diseases [10,12,24,25]. In pathogenic *E. coli*, virulence-associated large plasmids that are required to establish distinct disease phenotypes have been characterized using *in vitro* and *in vivo* studies [10,12–14,17,25]. Recently, it has been suggested that the plasmids may play a role in NMEC pathogenesis since most of the NMEC strains harbor plasmid-associated genes as compared to commensal *E. coli* [26]. *Escherichia coli* RS218 which was isolated from CSF of a neonate with meningitis in 1974 is considered as the prototype strain of NMEC. This strain has been used in the studies since then to identify the virulence traits that are particularly involved in NMEC pathogenesis [16]. Here, we determined and analyzed the complete nucleotide sequence of pRS218, a large plasmid of *E. coli* RS218, and studied its contribution to the NMEC pathogenesis.

The pRS218 sequence revealed a backbone typical to IncFIB/IIA-like plasmids in other pathogenic *E. coli* which possess both *repA* and *repA1* replicons [10]. In addition to the replication proteins, the constant region of the plasmid encodes proteins involving conjugal transfer (Tra locus) and plasmid stability/inheritance. The *tra* locus comprises 34.9 kb region containing 34 *tra* genes from *traM* to *finO* similar to F-like plasmids of *E.coli* and R100 plasmid of *Shigella* [27]. The plasmid SOS inhibition protein (PsiAB), plasmid stabilizing proteins StbAB and CcdAB, toxin-antitoxin proteins involved in post segregation killing are also present in the constant region that confers stability and inheritance of the plasmid in progeny cells. Parallel to these findings, we have observed that the curing of pRS218 is very difficult with chemical methods such as ethidium bromide and SDS treatment alone. Therefore, we mutated the *stbA* gene which has been identified as an essential gene for stable inheritance of IncF plasmids to achieve successful curing of pRS218 from *E. coli* RS218.

Genetic load region or the variable region of the pRS218 contains IS elements, virulence-associated genes, and several putative and hypothetical genes. The pRS218 contains 20 IS elements belonging to twelve different types. Previous studies have shown that IS-mediated recombination might play a major role in acquiring novel genes into plasmids thereby allowing the plasmid to act as a “pathogenicity island precursor” [10,12,14]. Interestingly, IS elements of pRS218 are located upstream or downstream of virulence/fitness-associated genes in genetic load regions providing further evidence for such speculation (Figure 1). Types of virulence or fitness genes in the genetic load region of pRS218 are depicted in Table 1 and are mainly located upstream and downstream of IncFIB replicon. Upstream to the IncFIB replicon, are the secreted copper-sensitivity suppressor proteins C and D (*scsC* and *scsD*). Copper is an essential trace element required for bacterial growth and it acts as a toxic compound if available in excess. Antibacterial properties of ionic copper have been studied and used in hospital settings to prevent nosocomial infections [28]. The pRS218 encoded *scsC* and *scsD* are identical to copper suppressor proteins in the genomic island GI-DT12 of *Salmonella enterica* subsp. *enterica* serovar Typhimurium str. T000240 which have been studied in relation to conferring copper resistance in recombinant *E. coli* carrying GI-DT12 providing a fitness advantage to the pathogen [29]. Additionally, this region encodes several iron acquisition proteins, hemoglobin receptors and a putative ABC transporter which may be involved in the survival of bacteria in an iron-limited milieu inside the host. Furthermore, pRS218 also encodes an enterotoxin called SenB, which has been found in enteroinvasive *E. coli* and S*higella* spp and accounts for 50% of their enterotoxic activities [30]. Interestingly, nucleotide blasting of *senB* sequence reveled that it is also present in the genomes of *E. coli* CE10 and the *Citrobacter koseri* which are associated with meningitis in newborns. Moreover, *senB* is located just downstream of *cjr* operon which is an iron- and temperature-regulated operon expressed only during the pathogenic process of *E. coli* suggesting that *senB* may be involved in NMEC pathogenesis [30]. A recent study reported that mutation of *cjr* area of pUTI89 (which is >99% similar to pRS218) significantly decreased bacterial invasion and intra-cellular bacterial community (IBC) formation in infected bladders [12]. However, the association of pRS218-encoded traits such as SenB in NMEC penetration of the intestinal epithelium and iron acquisition systems in NMEC survival within the host are yet to be identified. Other than these putative virulence-associated genes, many hypothetical proteins of unknown functions are present both upstream and downstream of IncFIB replicon.

Furthermore, we screened 59 pRS218 genes among 53 NMEC strains and fecal *E. coli* strains isolated from healthy individuals. A vast majority of pRS218-associated genes tested were overly represented among NMEC strains as compared to commensal *E. coli* (Table 3) suggesting a relationship between the presence of pRS218 genes and the NMEC pathotype. These overly represented genes included several hypothetical proteins and virulence-associated genes present in pRS218 such as copper sensitivity, iron acquisition, ABC transporter components, *traJ* and *senB*.

We also analyzed the sequence similarity and the evolutionary relationship of pRS218 with other NMEC plasmids, namely pECOS88 and pCE10A, and some other IncFIB/IIA plasmids of pathogenic *E. coli* (Figures 2 and 3). The pRS218 showed a remarkable sequence similarity to four plasmids found in *E. coli* associated with acute cystitis (pUTI89, pEC14_114, p1ESCUM, and pUMN146) and a plasmid present in an enteroinvasive *E. coli* (pECSF1) (Figure 2). The differences detected among pRS218, pUT89, pEC14_114 and pUMN146 revealed only SNPs and insertion of a *tetABCD* antibiotic resistance cassette in p1ESCUM and pECSF1 (Figure 2 and Table 4). However, the nucleotide sequence of pRS218 showed a marked difference from those of two NMEC plasmid sequences currently available in the public domain. For example, pECOS88 shares similarity only with *tra* locus, *repA* and *repA1* regions of pRS218 revealing that the genetic load regions of these plasmids harbor different putative virulence and hypothetical genes to those of pRS218. Compared to pECOS88, pCE10A plasmid showed a relatively higher nucleotide sequence similarity to pRS218 genetic load region containing the copper resistance-associated genes (*scsDC*), *cjrABC* and *senB*. However, pCE10A lacks the *tra* locus thereby making the plasmid incapable of conjugal transfer.

**Table 4:** **Point mutations and single nucleotide polymorphisms observed between pRS218 and pUTI89 sequences**

**pRS218 base position**	**pUTI89 base position**	**Point mutation type**	**pUTI89 base**	**pRS218 base**	**Gene name**
4956	4956	SNP	G	A	Intron
8972	8972	Indel	C	-	Putative membrane protein
17429	17429	Indel	-	C	Hypothetical Protein
17440	17439	Indel	-	C	Hypothetical Protein
17997	17995	SNP	A	G	Hypothetical Protein
19955	19953	SNP	C	A	Intron
39234	39232	Indel	A	-	Putative hemin receptor
39237	39235	Indel	T	-	Putative hemin receptor
51720	51718	SNP	G	T	Resolvase
53062	53060	SNP	C	T	Intron
64393	64391	Indel	C	-	*ycfA*
73197	73195	Indel	C	-	*psbl*
77808	77806	Indel	-	A	Intron
91272	91269	SNP	T	G	*trb*C

Among many capsular types of *E. coli*, K1 is the most common type associated with NM and according to previous studies, approximately 80% of NMEC possessed a K1 capsule [4,5]. Neonates acquire *E. coli* K1 mainly from the urogenital microflora of the mother. Although there are no studies done on the mechanisms that facilitate the vaginal epithelial colonization and survival of the NMEC strains in the urogenitary tract of women, it has been well documented that cystitis causing *E. coli* can survive and persist inside bladder epithelial cells as IBCs which is a dormant stage that becomes activated and shed when the immunity of the host is suppressed as is the case during pregnancy [26]. The same study has also indicated that the pUTI89 plasmid is essential for filamentation of IBCs which is the first event of reactivation of *E. coli* from the dormant state. A high degree of sequence similarity of pRS218 to other cystitis-associated plasmids and their close evolutionary relationship suggest that *E. coli* RS218 might use the same strategy to survive in the urogenitary tract. However, the ability of *E. coli* RS218 to invade bladder epithelial cells and to survive within the urogenitary tract remains to be investigated.

Pathogenesis of NMEC meningitis involves three main sequential events that are governed by the virulence potential of bacteria. These include initial colonization and invasion of gastrointestinal tract, survival and multiplication in blood, and invasion of BBB [5]. We examined the pathogenic potential of pRS218 to penetrate BBB *in vitro* and *in vivo* using hCMEC/D3 cells and a rat pup model of neonatal meningitis, respectively. Curing of pRS218 from *E. coli* RS218 did not show any effect on the growth rate revealing that differences observed between wild type and plasmid cured strains during *in vitro* and *in vivo* studies were not due to the differences in their growth rates (Figure 4C).

It is believed that the high level of septicemia is a prerequisite for the penetration of BBB by NMECs to establish neonatal meningitis [4]. We observed a higher incidence of septicemia among the rat pups infected with wtRS218 strain (84%) than the RS218_cured_ strain indicating that plasmid-encoded genes might be involved in developing septicemia. Iron is a major limiting factor that restricts the survival and multiplication of bacteria inside the host. The genetic load region of pRS218 encodes several high affinity iron acquisition proteins, hemolysin modulation factor and hemoglobin receptor which may be involved in iron acquisition. Interestingly, these genes were highly prevalent in NMEC strains as compared to fecal *E. coli* (Table 3). Furthermore, *in vitro* and *in vivo* study results clearly demonstrated that RS218_cured_ strain is far less capable of invading epithelial and endothelial cells as well as establishing meningitis in neonatal rat pups as compared to its wild type strain, suggesting that pRS218 might play a role in NMEC pathogenesis. The *traJ* which is present in pRS218 has been previously identified as a potential virulence trait in NMEC by signature-tagged mutagenesis and *in vitro* endothelial invasion assays [31]. The mutation of *traJ* was shown to be attenuated in terms of invasive ability to penetrate the BBB. However, more than 50% of the NMEC strains used in this study did not possess *traJ* even though the gene was more prevalent in NMEC than in fecal *E. coli* (Table 3). The present study demonstrated that the curing of pRS218 offered a greater attenuation to RS218 strain than did the mutation of *traJ* alone suggesting that addtionalpRS218 genes other than *traJ* might be involved in NMEC pathogenesis. Interestingly, as shown in Table 3, pRS218 carries several genes that encode hypothetical proteins which are also more prevalent in NMEC than in fecal commensal *E. coli*. Most gene prevalence studies carried out to identify potential virulence markers of NMEC have used already known virulence genes of other ExPEC and only a limited number of studies have attempted to explore novel traits that might be helpful in defining the NMEC pathotype [5,26,32]. Therefore, future studies aimed at delineating the mechanistic aspects of hypothetical proteins encoded by pRS218 and are more commonly occurring in NMEC than in fecal commensal *E. coli* may help to close the knowledge gaps pertaining to our understanding of NMEC pathogenesis.

Although RS218_cured_ strain was significantly attenuated in terms of *in vitro* (on the basis of bacterial invasion) and *in vivo* (on the basis of reduction in bacterial counts in CSF and blood from infected rat pups) assays as compared to the wild type strain, it was not completely avirulent. This finding suggests that the full virulence of *E. coli* RS218 requires both chromosomal and plasmid-located genes. Further studies including in depth analysis of RS218 chromosome will advance our understanding of NMEC pathogenesis.

## Conclusions

Incomplete understanding of NMEC pathogenesis is a major hindrance that has been identified and pointed out by many scientists particularly in relation to formulation of novel therapeutic and prevention strategies for neonatal meningitis. The plasmid pRS218 in NMEC RS218 strain belongs to IncFIB/IIA subset of virulence plasmids in pathogenic *E. coli*. These plasmids harbor many virulence traits that are required for bacterial survival inside the host. The nucleotide sequence of pRS218 showed a greater similarity to the plasmids of *E. coli* associated with acute cystitis than the plasmids from NMEC. However, the prevalence of pRS218 virulence-related genes was significantly higher in NMEC strains tested than fecal commensal *E. coli*. We have also demonstrated that the pRS218 is involved in NMEC pathogenesis using both *in vivo* and *in vitro* experiments. Future studies on pRS218 transcriptome analysis, identification of plasmid-located genes responsible for current observations and in-depth analysis of *E. coli* RS218 whole genome will likely broaden our knowledge of NMEC pathogenesis.

## Methods

### Bacterial strains and media

The prototype NMEC strain *E. coli* RS218 (O18: H7: K1) and NMEC strain EC10 (O7: K1) were kindly provided by Dr. James Johnson (Department of Medicine, University of Minnesota, Minneapolis, MN). Both *E. coli* RS218 and EC10 strains have been isolated from cerebrospinal fluid of neonates diagnosed with bacterial meningitis (15). A total of 51 NMEC strains which were isolated from neonatal meningitis cases were also obtained from Dr. K.S. Kim (School of Medicine, John Hopkins University, Baltimore, MD) and 49 fecal *E. coli* strains isolated from feces of healthy individuals were obtained from the *E. coli* Reference Center (Pennsylvania State University, University Park, PA). All *E. coli* were stored in Luria Bertani broth (LB) at -80°C until further use. Bacteria were grown in MacConkey agar or LB broth. All bacteriologic media were purchased from Becton, Dickinson and Company (BD), Sparks, MD.

### Plasmid isolation, sequencing, assembly and annotation

Sequencing of pRS218 was performed as a part of a project that sequenced the whole genome of *E. coli* RS218. The genomic DNA including the plasmid DNA was extracted using phenol-chloroform method as described previously [33]. The DNA preparation was further cleaned using Genomic Tips (Qiagen, Valencia, CA) [33]. Whole genome sequencing was performed using Ion Torrent PGM Technology (Life Technologies, Carlsbad, CA) at the Genomics Core Facility (Pennsylvania State University, University Park, PA). After initial *de novo* assembly of short reads using SeqManNGen 10 (DNASTAR Inc, Madison, WI), plasmid contigs were identified using BLAST algorithm (Blastn; www.ncbi.nlm.nih.gov) and subsequently aligned to the sequence of the reference plasmid, pUTI89 [GenBank:CP000244]. Gap closure was performed using primer walking into the gaps with the LongRange PCR Kit (Qiagen). The complete sequence of the plasmid was annotated using Rapid Annotation using Subsystem Technology (RAST) [34].

### Comparative genomics and phylogenetic analysis

Comparative genomics of pRS218 with closely related IncFIB/FIIA plasmids of other *E. coli* was performed using Mauve 3.2.1 genome alignment web tool (http://gel.ahabs.wisc.edu/mauve/) [35]. An evolutionary relationship of 24 plasmids belonging to the IncFIB/FIIA group based on *repA1* gene sequence was performed using the neighbor-joining method. A neighbor joining tree was constructed by using the MEGA4 web tool (http://www.megasoftware.net/mega4/mega.html) [36,37].

### Analysis of plasmid profiles of NMEC strains

Extraction of large plasmids from NMEC strains was performed using an alkaline lysis method described previously [33]. In brief, 1 ml of overnight culture of each *E. coli* strain was subjected to alkaline lysis using 10% sodium hydroxide followed by phenol-chloroform extraction of plasmid DNA. Plasmid profiles of NMEC strains were evaluated by electrophoresis on a 0.7% agarose gel containing 0.5 μg/ml ethidium bromide.

### Evaluation of prevalence of selected pRS218 genes in other NMEC and fecal *E. coli*

Specific polymerase chain reactions (PCRs) were performed to determine the presence of selected gene coding regions (n = 59) of pRS218 in other NMEC and fecal *E. coli* strains. Primers were designed using the Primer 3.0 web tool (http://bioinfo.ut.ee/primer3-0.4.0/) (Table 5). PCR amplifications were performed using crude DNA extracted by the rapid boiling method [38]. The PCR mixture contained 1 U of *Taq* polymerase (Qiagen), 1× *Taq* polymerase buffer, 3.5 mM MgCl_2_, 125 μM each deoxynucleotide triphosphate (dNTP) and150 nM each primer pair. PCR conditions were as follows: 1 cycle of 95°C for 1min, followed by 30 cycles of 95°C for 30 s, 55°C for 30 s, and 72°C for 1.5 min, and a final extension at 72°C for 10 min. Amplicons were visualized on a 1.5% agarose gel containing 0.5 μg/ml ethidium bromide.

**Table 5:** **Primers used for the screening of pRS218 genes among neonatal meningitis causing** ***E. coli*** **and fecal commensal** ***E. coli*** **strains**

**Coordinates in pRS218**	**Gene name**	**Predicted function**	**Primer F (5′-3′)**	**Primer R (5′-3′)**	**Product size (bp)**
4107- 4265	pRS218_007	Copper sensitivity	gagacgttgagcaccaatctg	accgccagtttttctttcac	140
4255- 4761	pRS218_008	Copper sensitivity	catacgctggacggggaaac	gacgctctccccttccgact	143
4998- 5759	pRS218_010	Na + traslocation	atcaatgatggtgctttgtgtc	ccggtaactggaatgataacct	378
6052-7992	pRS218_013	Iron permease	gtgttcgagaacctggaagg	cggttttgtctgagggacat	401
8033- 8560	pRS218_014	Iron transport	ctgtcaccatgaatgaaatgga	ctcacatcaaacggtttccac	400
8664- 10043	pRS218_015	Membrane protein	tcgtgacggtaaactgcatc	gccgccatagctgtatttgt	400
10046- 11329	pRS218_016	ABC transporter	aaggggtggtgatcgataaaat	catacagcacctccacaggata	399
11319- 12449	pRS218_017	Membrane protein	aggtcaccggtagctggatt	atcgagaccagtcccatcag	400
12454- 13149	pRS218_018	ABC transporter	gttccatttgatcccgttctta	acccagatatttaccgtgttgc	379
13136- 13621	pRS218_019	Putative thioredoxin precursor	gcgggtgtaaagaagaaaagc	agacggcttacgcataccc	401
13646- 14131	pRS218_020	Hypothetical protein	atagcgcaactgcttcacacta	acgttccgtatcgacaaattct	303
14253- 14702	pRS218_022	Glucose-1-phosphatase	agacaacgccggaaggttat	tttcctgatgatgtaccggaat	354
14677- 14997	pRS218_023	Glucose-1-phosphatase	acgatggacccaacgtttaat	ataggctgattcgatgtgtttg	311
18173- 17826	pRS218_031	Hypothetical protein	attgccctgatggacagc	gtggcagccggttaacttt	301
20251-20775	pRS218_034	Colicin immunity	ttaataatatgtggtggggatgg	atgaaaacagtacccgtataaacagc	250
21065-21982	pRS218_035	ColicinJ production	tggcttattcaaaatttgctcat	tgcatagatatgatggtttcacg	350
21990-22766	pRS218_036	ColicinJ production	ctgattttccttgcgtttatctg	agcctttatcttacgaggtggac	294
22935- 25196	pRS218_038	ColicinJ production	tatgatgcaggttttgcttttg	tggcatcatgttgagcttattc	393
25265- 26440	pRS218_039	Enterotoxin	gcagattcgcgttttgagca	cggatctttcaacgggatgg	302
28517- 27762	pRS218_042	Hypothetical protein	tgacgctatgcaatgaagaact	tgacatagccaagatcatccac	399
38291- 37500	pRS218_056	Hypothetical protein	cgtccacggattatgtctataaaac	gtatgacgggatgatttcagataac	373
40184- 38298	pRS218_057	ColicinJ production	ctgtggataacagcctcatcaa	atgttaaccgggtagcttttca	301
43799-42630	pRS218_060	Hypothetical protein	ctcttccccatggcctttat	accccatactgcattggaaa	600
46748- 46975	pRS218_063	Hypothetical protein	tggatcctttgttgatcattcat	cctgtaaagacagacttcagaaaaa	224
48251- 47610	pRS218_064	Hypothetical protein	tcgacctaacccttgatcagtt	tatagcgacaggatggacagtg	385
52321- 52046	pRS218_073	Hypothetical protein	cagccagcaagcattaaaca	gctcaagggctactctgacg	276
53188- 54159	pRS218_074	Stability protein StbA	ttgtcgcaaaactcatttcg	cgaccagacgagaaaacaca	400
56513- 56265	pRS218_079	Hypothetical protein	cgcattgaaattcttttcgac	tcgtcctgccagatttcttc	249
56648- 57166	pRS218_080	Unknown	gtgttcgtgatctcgtttcgta	ttgcccactttcttaatcttcc	351
58824- 59654	pRS218_082	Hypothetical protein	acaaatgaaggtattcagctgtttc	cgacagtacgttgtcacacagac	372
60445-59648	pRS218_083	Transposase	gcttcgggaacgctgtaacg	agaaggctgcggtgctgaag	414
61858- 62169	pRS218_086	Hypothetical protein	ttttccggtaaaggatgtcg	gtctttctgacggcaaggctat	223
62245- 62928	pRS218_088	Adenine-specific methyltransferase	cggtgatgttaatgatgactgg	gtgtgaagctctcaatcagtgg	356
62929- 63150	pRS218_089	Cytoplasmic protein	ctatgccggacacgaaaaac	gaagcaggaatccagttcca	208
63230- 63598	pRS218_090	Hypothetical protein	gttatctggtccccggaaga	cattcacgtttccacaatgc	254
63643- 64614	pRS218_091	Hypothetical protein	atgaatgaaatgctgaatgcac	catcttctgccacctggtaact	406
63643-64614	pRS218_091	Hypothetical protein	cgcctggtggtgaaggaaag	gaccacctcccgcagaacac	236
64828- 65253	pRS218_092	Putative antirestriction protein	gttgaagagtgcgaccgtct	agtcaagtgccgcgtaaatc	400
65300- 65722	pRS218_093	Phage protein MubC	catccgcgatgtactggatac	ctgtaacacaacgtccattgct	373
65719- 65910	pRS218_094	Hypothetical protein	cacagaaacccgcgaaat	ctgtttctgctgccctgtaag	177
66381- 65887	pRS218_095	Hypothetical protein	cttacatcccggcgtcgt	cctgatgttatgtttctgtggttact	256
67155- 68516	pRS218_099	Hypothetical protein	tatggcaaaactcatcagcagt	gtaatttggcgttgtgactgaa	385
68563- 69126	pRS218_100	Hypothetical protein	tctcagctttttgtgagtcctg	aaaacggtaacagcttctcctg	400
70556- 70789	pRS218_105	Cytoplasmic protein	gcgaatatttcagaatacttcagg	aattccggatgacatggttc	213
70848- 72806	pRS218_106	Hypothetical protein	agtgtgaggaatctgacctgct	taatgtttacattccaggctgattt	400
72861- 73271	pRS218_107	Adenine-specific methyltransferase	ataccatgaacgcacaggaata	ggatgatgtcgttaacgctgta	371
74286- 74444	pRS218_109	Hok/Gef cell toxic protein	atgaaactaccacgcagctctc	taccggattcgtaagccatga	154
75004- 74681	pRS218_110	Hypothetical protein	gcgttgcgccttacatcc	tcacatcaccttccctttgatt	314
75360- 75647	pRS218_113	Hypothetical protein	gagtacccgaaatatccacgtt	taatctgacgcaggaactgttt	251
75360-75647	pRS218_113	Hypothetical protein	tgggggctgaaaaccagaga	accgaaggcacgaactgcat	531
75691- 76587	pRS218_114	Unknown	tcggtattttccggtgataaac	ataacctgcccgacaatatcac	359
77473- 76883	pRS218_116	X polypeptide	aggccgggattacaaaatagat	ccggtataaatccggtaaacct	354
78394 79080	pRS218_118	TraJ/conjugal transfer	caatggggcttttattgaactc	tgaccaacacccagcatataaa	369
85396- 85614	pRS218_131	Hypothetical protein	tgcatacctttatttttcttgtgc	tcagtgtatccatcacgttgttc	210
89620-90612	pRS218_136	TraU/conjugal transfer	ttccttctcgccggtcatgt	ccagcgagagcgggaaaata	111
105274- 110544	pRS218_154	TraI/conjugal transfer	gcgatgcggtcagtgttctg	ggacagccgttcatcctgct	190
111369- 112229	pRS218_156	Dienelactone hydrolase	tctggttaccggagagatgaat	agtaccagaagcaacagcatca	343
113415- 113939	pRS218_159	Hypothetical protein	gtgccatttatctgatatggagaat	tctgtgttgtactgctcatataccc	387
113985- 114194	pRS218_190	Hemolysin expression modulating protein	caaaacaggaatggctgtatca	tatttccatatctcttttggtatcctg	190

### Plasmid curing and complementation

The plasmid stability gene, *stbA* of pRS218 was mutated by using a phage lambda Red recombinase system to facilitate plasmid curing [39]. Briefly, the chloramphenicol resistance cassette (*cat*) was amplified with PCR by using pKD3 plasmid as the template and primers consisted of 36 nucleotides extensions at 5′ and 3′ ends of *stbA* (forward primer 5′- ATG AAC GTA TAC TGC GAT GAT GGT TCA ACA ACA ATC GTG TAG GCT GGA GCT GCT TC-3′ and reverse primer 5′-TAC TCC TCT TTG AAA GCC GCG ATA GCT TCA ACC AGT CAT ATG AAT ATC CTC CTT AG-3′). Amplified product was gel purified (MiniElute Kit, Qiagen) and electroporated to *E. coli* RS218 carrying the Red helper plasmid, pKD119. Mutants (RS218:pRS218∆*stbA*::*cat*) were selected for chloramphenicol resistance and confirmed by PCR using primers specific to *stbA*. Plasmid curing was done as described previously by 10% sodium dodecyl sulfate treatment [40]. Plasmid curing was confirmed by comparing the plasmid profile of the cured strain with the parent strain. The isolate which did not possess the plasmid was further verified for curing by PCR amplification of 5 genes or ORFs, *senB* (forward primer 5′- GCA GAT TCG CGT TTT GAG CA-3′ and reverse primer 5′- CGG ATC TTT CAA CGG GAT GG-3′), *scsD* (forward primer 5′- CAT ACG CTG GAC GGG GAA AC-3′ and reverse primer 5′-GAC GCT CTC CCC TTC CGA CT-3′), *traU* (forward primer 5′- TTC CTT CTC GCC GGT CAT GT-3′ and reverse primer 5′- CCA GCG AGA GCG GGA AAA TA-3′), *transposase* (forward primer 5′- GCT TCG GGA ACG CTG TAA CG-3′ and reverse primer 5′- AGA AGG CTG CGG TGC TGA AG-3′)*, pRS218_113* (forward primer 5′- TGG GGG CTG AAA ACC AGA GA-3′ and reverse primer 5′- ACC GAA GGC ACG AAC TGC AT-3′), and *ycfA* (forward primer 5′- CGC CTG GTG GTG AAG GAA AG-3′ and reverse primer 5′- GAC CAC CTC CCG CAG AAC AC-3′) of pRS218. Isolates that did not possess all of the five genes/ORFs were considered to be cured of pRS218.

The plasmid complementation was performed using conjugation as described previously [41]. The main obstacle for complementation was the absence of an antibiotic resistance marker in pRS218 which could have been used for subsequent selection. Therefore, pRS218 was first tagged with *cat* using the one step inactivation method [39]. Briefly, the *cat* was amplified using pKD3 plasmid and primers consisted of 36 nucleotides extensions at 5′ and 3′ ends of a putative noncoding region of pRS218 located between base pairs 591 and 831 in the plasmid sequence (Forward primer 5′-CGC CTT CGC GTT GCT CAG TTG TCC AAC CCC GGA AAC GTG TAG GCT GGA GCT GCT TC-3′ and reverse primer 5′-CTC CTC AAT ACT CAA ACA GGG ATC GTT TCG CAG AGG ACA TAT GAA TAT CCT CCT TAG-3′). Purified PCR product was electroporated to *E. coli* RS218 carrying the Red helper plasmid pKD119 to construct the pRS218::*cat*. The temperature sensitive pKD119 plasmid was removed from pRS218::*cat* by growing at 42°C followed by screening for tetracycline sensitivity. The *E. coli* RS218 carrying pRS218::*cat* was then used as the donor to perform mating experiments. *Escherichia coli* DH5α was used as an intermediate recipient to transfer pRS218::*cat* from the donor strain to the recipient plasmid-cured strain.

### Bacterial growth curve

Bacteria were grown in LB broth at 37°C with shaking overnight. Cultures were diluted to 1:100 with LB broth, tissue culture medium or M9 medium with 10 μg/ml niacin and incubated at 37°C with shaking. Optical density at 600 nm (OD600) was taken in triplicate for every 20 min for 6 hrs. The OD values from each time point were averaged and graphed to obtain a growth curve.

### *In vitro* invasion assay

Invasion assays were performed using hCMEC/D3 cells provided by Dr. Weksler B, Cornell University, NY. The hCMEC/D3 cells were grown in endothelial basal medium (Lonza, Walkersville, MD) containing 5% fetal bovine serum (PAA The Cell Culture Company, Piscataway, NJ), 1.4 μM hydrocortisone (Sigma-Aldrich, St. Louis, MO.), 5 μg.ml^−1^ acid ascorbic (Sigma), 1% chemically defined lipid concentrate (Gibco, Carlsbad, CA), 10 mM HEPES (PAA The Cell Culture Company), and 1 ng.ml^−1^ human basic fibroblast growth factor (Sigma), The invasion assay was performed as described previously [32]. Briefly, endothelial cells were seeded at about 1 × 10^5^ cells per well in 12-well tissue culture plates (Corning Life Sciences, Manassas, VA.) coated with rat collagen (R&D Systems, Trevigen, Gaithersburg, MD) and incubated at 37°C with 5% CO_2_ in a humid chamber. Once the monolayer was confluent, it was washed with phosphate-buffered saline (PBS, pH 7) and incubated with the cell culture medium containing bacteria at a multiplicity of infection (MOI) of 100 for 2 hrs at 37°C with 5% CO_2_ to allow cellular invasion [32]. The extracellular bacteria were eliminated by incubation of the monolayers with a culture medium containing gentamicin (100 μg/ml) for 1 h. The monolayers were washed three times with PBS and lysed with 0.1% Triton X-100. The intracellular bacteria that were released during cell lysis were enumerated by plating on LB agar plates. Invasion frequencies were calculated by dividing the number of invaded bacteria by the initial inoculum and expressed as a percentage relative to the invasion frequency of wtRS218. The assays were performed three times in triplicate and student’s *t* test was used to compare the groups.

### Neonatal rat meningitis model

Five-day-old Sprague-Dawley out-bred rat pups (n = 10) were used in each experimental group. Rat pups were injected with approximately 200 CFU (range160 to 210 CFU) of *E. coli* (wtRS218 and RS218_cured_) by the intraperitoneal route. For the negative control group, PBS was injected intraperitoneally. Mortalities of rat pups in each group were monitored for 24 hrs post-inoculation. The pups that survived were euthanized 24 hrs post-inoculation to collect blood, cerebrospinal fluid (CSF) and brain tissues. For bacterial enumeration, blood was collected by intra-cardiac puncture and plated on MacConkey agar to detect septicemia. Cerebrospinal fluid was collected by cisternal puncture, and plated on MacConkey agar to demonstrate meningitis. Brain tissues collected from each group were fixed in 10% neutral-buffered formalin, routinely processed for histopathology, stained with haematoxylin-eosin, and examined for lesions consistent with bacterial meningitis. Experiments were done in triplicates and the paired *t* test was used to compare the experimental groups.

### Ethics statement

Protocols involving rat experiments complied with federal guidelines and the policies of the Institutional Animal Care and Use Committee (IACUC) of the Pennsylvania State University (University Park, PA). Both NMEC and HFEC isolates, in their entirety, were collected for purposes other than this study and were given without any Health Insurance Portability and Accountability Act (HIPAA) identifiers by Dr. K.S. Kim (John Hopkins University, Baltimore, MD).
